# Accelerated design of nickel-cobalt based catalysts for CO_2_ hydrogenation with human-in-the-loop active machine learning†

**DOI:** 10.1039/d4cy00873a

**Published:** 2024-09-10

**Authors:** Yasemen Kuddusi, Maarten R. Dobbelaere, Kevin M. Van Geem, Andreas Züttel

**Affiliations:** a Laboratory of Materials for Renewable Energy (LMER), Institute of Chemical Sciences and Engineering (ISIC), Basic Science Faculty (SB), École Polytechnique Fédérale de Lausanne (EPFL) Valais/Wallis, Energypolis Rue de l'Industrie 17 1951 Sion Switzerland yasemen.kuddusi@epfl.ch; b Empa Materials Science & Technology 8600 Dübendorf Switzerland; c Laboratory for Chemical Technology, Department of Materials, Textiles and Chemical Engineering, Ghent University Technologiepark 125 9052 Gent Belgium

## Abstract

Thermo-catalytic conversion of CO_2_ into more valuable compounds, such as methane, is an attractive strategy for energy storage in chemical bonds and creating a carbon-based circular economy. However, designing heterogeneous catalysts remains a challenging, time- and resource-consuming task. Herein, we present an interpretable, human-in-the-loop active machine learning framework to efficiently plan catalytic experiments, execute them in an automated set-up, and estimate the effect of experimental variables on the catalytic activity. A dataset with 48 catalytic activity tests was compiled from a design space of Ni–Co/Al_2_O_3_ catalysts with over 50 million potential combinations in only eight iterations. This small dataset was found sufficient to predict CO_2_ conversion, methane selectivity, and methane space–time yield with remarkable accuracy (*R*^2^ > 0.9) for untested catalysts and reaction conditions. New experiments and catalysts were selected with this methodology, leading to experimental conditions that improved the methane space–time yield by nearly 50% in comparison to the previously obtained maximum in the dataset. Interpretation of the model predictions unveiled the effect of each catalyst descriptor and reaction condition on the outcome. Particularly, the strong predicted inverse trend between the calcination temperature and the catalytic activity was validated experimentally, and characterization implied an underlying structure–performance relationship. Finally, it is demonstrated that the deployed active learning model is excellently suited to predict and fit kinetic trends with a minimal amount of data. This data-driven framework is a first step to faster, model-based, and interpretable design of catalysts and holds promise for broader applications across catalytic processes.

## Abstract 2

The effect of catalyst synthesis and reaction conditions on catalytic activity were accurately predicted with an interpretable data-driven strategy. The method is demonstrated for CO_2_ methanation and is extendable to other catalytic processes.

## Graphical abstract

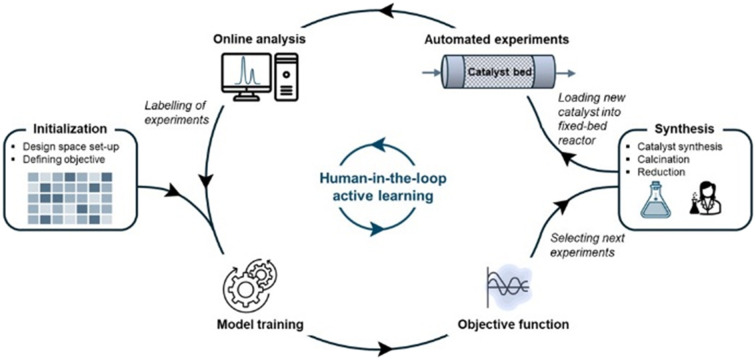

## 1. Introduction

CO_2_ plays a key role in the many carbon cycles that nature exhibits and exploits to store energy.^1^ It remains one of the main societal challenges to mimic the natural energy storage capacities by technology and secure the future energy supply while minimizing CO_2_ emissions.^1^ So-called chemical energy carriers provide a promising way of storing renewable energy. In such processes, CO_2_ is used as feedstock and catalytically reduced to synthetic hydrocarbons in which the energy is stored in the chemical bonds.^2,3^ In particular, the Sabatier or methanation reaction (eqn (1)), which converts CO_2_ into CH_4_, is of paramount importance. This reaction has a high technological readiness level due to the existing natural gas grid infrastructure and realizations that transitioned methanation endeavors from lab to pilot and industrial scale.^4–8^ Nevertheless, catalyst design and reaction modeling improvements are still heavily needed to make it a mature energy storage method.^9^

1 CO_2_ + 4H_2_ ⇋ CH_4_ + 2H_2_O Δ*H*_298 K_ = −165.0 kJ mol^−1^

The exothermic methanation reaction is thermodynamically favored at low temperatures and high pressure. However, a catalyst and high temperatures (typically 300 to 500 °C (ref. 10)) are required to overcome the kinetic barrier and achieve sufficient reaction rates.^11^ At such temperatures, CO_2_ methanation can also be accompanied by the undesired reverse water-gas-shift reaction (eqn (2)).

2 CO_2_ + H_2_ ⇋ CO + H_2_O Δ*H*_298 K_ = +41.0 kJ mol^−1^

The active phase of a methanation catalyst is usually based on a transition metal such as Ru, Rh, Pd, Pt, Ni, or Co.^10,12,13^ Considering the aspired industrialization of the methanation process, the metal price is an important incentive that explains the popularity of Ni-based catalysts.^9,11,12,14^ Next to the price, Ni is selective towards CH_4_. The activity of a Ni-based catalyst depends on synthesis parameters such as the support type, Ni loading, synthesis method, and the presence of a second metal. Various support materials with high surface area have been investigated for Ni-based methanation catalysts, such as Al_2_O_3_, CeO_2_, or TiO_2_, as well as metal–organic frameworks and zeolites. Al_2_O_3_ is the most commercialized support, given its low price, thermal stability, high surface area, and porous structure.^9^

Ni/Al_2_O_3_ catalysts, while susceptible to deactivation, have shown some enhancements with the introduction of additional metals. Especially Co, also a non-noble metal, has sparked interest as a Ni promoter because of its beneficial effect on Ni dispersion, stability, reducibility, coke deposition, and activity.^9,15–17^ As altered catalyst synthesis recipes and reaction conditions may change the kinetics and mechanistic pathway of CO_2_ methanation, it can be assumed that the rate expressions will differ as a result of these alterations.^12^ Ideally, the design of catalysts and the exploration of reaction conditions should be viewed as a unified, multi-dimensional challenge to guarantee the best possible reactor design and operation across various scales.

Optimization of heterogeneous catalysts is a high-dimensional problem that encompasses a vast space of different material compositions, preparation methods, and reaction conditions. The extent of the search space results in the number of experiments typically being constrained by the project time and materials cost, even when automated high-throughput setups are available.^18,19^ Therefore, the scope of many studies is limited to a single catalysts modeled following a “postdictive” approach.^20^ That is, all necessary experiments must be performed to fit power-law rate expressions. This approach has the benefit that it is interpretable, can be used in reactor design, and is easy to solve numerically, but it requires a large number of experiments.^21^ Therefore, a shift towards “predictive” modeling, where catalytic activity can be estimated before performing any costly experiments, is heavily desired.

In recent years, machine learning tools have increasingly been used to extract information from kinetic catalytic data and provide predictive insights into catalyst action.^22–26^ Yılmaz *et al.*^27^ published a purely data-driven study in which they applied random forests to analyze the methanation reaction using data from 100 publications. While this kind of predictive approach can provide new insights, it is also limited to simple output predictions and impedes reverse design of catalysts because of the lack of standardization in published catalytic data. Indeed, catalytic data can be categorized into various types, of which synthesis and kinetic catalytic data play a central role.^28^ Subtle differences in synthetic procedures (*e.g.*, calcination temperature, reduction time) reported in different laboratories can drastically influence the reproducibility of reported reaction outcomes. Therefore, it is essential to compare heterogeneous catalysts under identical conditions to create reliable predictive models.^29,30^ In addition, direct comparison between various catalyst reports is hampered by missing reaction metrics, such as space–time yields or turnover frequencies.^31–33^

The scarcity of standardized catalytic data and the large dimensionality of catalytic research problems have especially boosted the application of active machine learning algorithms, which integrate machine learning and the design of experiments (DoE).^34^ Bayesian optimization and active learning are common strategies within active machine learning for optimizing experimental planning.^35^ The general workflow of these strategies consists of iteratively computing a surrogate model of the objective function. Then, an acquisition function is created to select the next point with the predicted posterior distribution. This acquisition function can be used to balance exploration and exploitation of the search space. The difference between active learning and Bayesian optimization lies in the research objective. While Bayesian optimization aims to find an extremum of the black-box objective function, it is in active learning the goal to optimize the performance of the surrogate model and create a generalized model over the entire design space. A recent work by Ramirez *et al.*^36^ demonstrates the use of Bayesian optimization and a high-throughput screening platform to optimize heterogeneous catalysts in eight iterations for the reduction of CO_2_ to methanol. In that work, up to 24 catalysts were synthesized per iteration in a nearly fully automated way. However, in the absence of high-throughput synthesis and reaction platforms, parallel querying is typically not possible. Sequential querying, on the other hand, is constrained by experimental specifications. For example, a reactor can ramp up the temperature but requires more time to cool down. Moreover, catalyst synthesis is a time-consuming step that is often investigated with categorical variables, rather than by iterative synthesis of suggested catalysts.^37^ Therefore, limited to a single reactor, Ureel *et al.*^38^ restricted their active learning study for the catalytic pyrolysis of plastic waste to two pre-selected catalysts. We addressed these constraints in our work by introducing batch acquisition in a single reactor with multiple suggested reaction conditions per queried catalyst.

Active machine learning approaches make use of black-box regression algorithms (*e.g.*, Gaussian processes) so that the relationship between input and output cannot be interpreted.^39^ On the other end of the model spectrum are glass-box models, such as microkinetic models or symbolic regression models, that have interpretability as an inherent feature.^40^ A compromise lies in the development of grey-box models to make black boxes interpretable.^29^ Recent examples have illustrated the use of grey-box models to speed up information extraction from kinetic catalytic data. Suvarna *et al.*^41^ used black-box ensemble methods followed by *a posteriori* feature-importance analysis for the CO_2_ hydrogenation reaction to methanol. Such analysis aims to add understanding to the effect of input variables on the output and was also demonstrated, among others, for dry reforming of methane^42,43^ and CO_2_-assisted propane dehydrogenation.^44^ Using hydrodeoxygenation as a model reaction, Mendes *et al.*^45^ created an automated gray-box tool to recognize patterns in kinetic catalytic data. While it is inevitable that data science and machine learning will become a standard tool for reaction engineers, new and reliable methodologies need to be developed to integrate these new tools into an experimental campaign.

The aim of this work is to provide a robust, interpretable, and efficient methodology that integrates data science and experimental reaction engineering, using the screening of catalysts and reaction conditions for the CO_2_ methanation reaction as a case study. Our approach includes a novel human-in-the-loop active learning workflow to plan, execute, and analyze catalytic experiments so that an informative dataset can rapidly be generated in a single reactor system without the need for a high-throughput platform. To achieve this, we establish an automated reaction platform that incorporates online chromatographic analysis and an active learning tool. We explore various machine learning models to predict the catalytic activity for new sets of experimental variables and find good agreement between predictions and experiments. Additionally, it is demonstrated that the underlying relationships between the experimental variables and the catalytic activity are learned by the machine learning model and can again be linked with physical insights. This study provides a data-driven workflow to accelerate experimental catalytic research and that is deployable to other catalytic systems and reactions.

## 2. Materials and methods

### 2.1. Experimental design space

The evaluation of the catalytic CO_2_ methanation is limited to an experimental design space consisting of 7 parameters, which are given in Fig. 2. These design parameters are grouped into 3 input categories: the reaction conditions, catalyst properties, and catalyst treatment conditions. The possible reaction conditions include temperatures between 523 and 773 K, pressures between 1 and 10 bar, and gas-hourly space velocities between 3300 and 26 400 ml h^−1^ g_cat_^−1^. The catalyst properties define the composition of the catalyst, which is limited to Al_2_O_3_-supported catalysts. Two metals, Ni and Co, are selected as primary base and promoter because of their large commercial availability at low cost. The maximal Ni and Co loading are 25 and 10 wt%, respectively. An experimental step size of 1 wt% is considered for the metal loadings. The catalyst treatment is limited to catalysts prepared *via* the incipient wetness impregnation method. The calcination and reduction temperature are defined in steps of 50 K between 623 K and 923 K. Multiplying the number of selectable experimental variables leads to a total of 54 654 600 accessible configurations for the experimental design space.

### 2.2. Catalyst preparation

Ni/Al_2_O_3_, Co/Al_2_O_3_, and Ni–Co/Al_2_O_3_ catalysts with different Ni and Co weight percentages were synthesized by the incipient wetness impregnation method. The notation of the catalysts will be *x*Ni–*y*Co/Al_2_O_3_-*T* where *x* and *y* are the weight percentages of nickel and cobalt loading respectively and *T* is the calcination temperature. The desired loading was achieved by impregnation of aqueous solutions of Ni(NO_3_)_2_·6H_2_O (Sigma-Aldrich) and Co(NO_3_)_2_·6H_2_O (Sigma-Aldrich) onto γ-alumina pellets (Alfa Aesar) with a pore volume of 1.20 cm^3^ g^−1^. The nominal metal loading in the catalyst was varied for Ni from 0 to 25 wt% and for Co from 0 to 10 wt%. The impregnated catalyst was dried for 12 h at 383 K prior to calcination in static air for 4 h using a heating rate of 5 K min^−1^ at various temperatures between 623 to 923 K. The calcined catalysts were reduced under H_2_ flow for 2 h at various temperatures from 623 to 923 K.

### 2.3. Catalyst characterization

Nitrogen sorption at 77 K was carried out using the Belsorp Max II instrument. The total surface area was determined via the Brunauer–Emmett–Teller (BET) method. X-ray diffraction (XRD) patterns were obtained with a Bruker D8 Advance instrument using Cu Kα radiation (*λ* = 0.154 nm) at 40 kV and 40 mA. The data were acquired in the 2*θ* range of 10–80°, with an angular step size of 0.025° and a counting time of 1.5 s per step. The dried catalyst (∼20 mg) was put inside an alumina crucible to conduct thermogravimetric analysis (TGA) using a Netzsch TG209 F1 instrument under a dry air flow of 10 mL min^−1^ with 20 mL min^−1^ N_2_ as the protective gas from 30 to 700 °C at a ramp rate of 5 °C min^−1^. High-angle annular dark-field scanning transmission electron microscopy (HAADF-STEM) imaging and energy-dispersive X-ray spectroscopy (EDX) were performed using a Thermo Fisher Scientific Tecnai Osiris transmission electron microscope in scanning mode at an acceleration potential of 200 kV. Morphological analysis was conducted with a FEI Teneo 250 scanning electron microscope at 1.0–5.0 kV and a working distance of 2.5–5.0 mm. X-ray photoelectron spectroscopy (XPS) measurements were carried out on an Axis Supra instrument (Kratos Analytical) using the monochromated Kα X-ray line of an aluminum anode, with the pass energy set to 40 eV. The binding energy scale was referenced at 284.8 eV using the CC/CH component of the C1s orbital. Peak fitting was performed using CasaXPS, employing the Shirley method for background subtraction.

### 2.4. Catalyst evaluation

The catalytic performance of the catalysts was evaluated in a fixed-bed tubular reactor (8 mm O.D.) that was heated in a tube furnace. The reactor temperature and catalyst bed temperature were measured by thermocouples and all gas flowrates were monitored by mass flow controllers. The experimental system was monitored and controlled by LabVIEW. The diagram of experimental set-up is given Fig. S3.1.†

Prior to the reaction, 200 mg catalyst was placed in the reactor within quartz wool. The catalyst was then reduced in 5% H_2_ in N_2_ flow at the desired reduction temperature for 2 hours. The molar ratio of CO_2_ to H_2_ was 1 : 4 in the feed in which N_2_ was used an internal standard. The tubing in which the outlet gasses were flowed through was heated to 393 K and passed through a condenser to remove water. For automating the entire experimental process without user supervision, the Laboratory Virtual Instrument Engineering Workbench (LabVIEW) platform was used.

### 2.5. On-line product analysis

The composition of the outlet gas products was analyzed on-line using a gas chromatograph (SRI Instruments Multi Gas Analyzer 1) equipped with a silica gel and molecular sieve 13x column as well as a thermal conductivity detector (TCD) and a flame ionization detector (FID). He was used as the carrier gas. The microreactor unit which enables the product analysis is directly integrated into the computational workflow based on LabVIEW and Python. The product analysis is directly integrated into the computational workflow. The raw TCD and FID data are exported in ASCII format. Subsequently, the data is processed in Python using SciPy for peak detection and NumPy for peak integration. Peak areas were automatically quantified using calibration curves so that catalytic activity was reported in terms of CO_2_ conversion (*X*_CO_2__), selectivity towards CH_4_ (*S*_CH_4__) and CO (*S*_CO_), yield of CH_4_ (*Y*_CH_4__), and space–time yield of CH_4_ (STY_CH_4__). The expressions given in eqn (3)–(7) were used.3
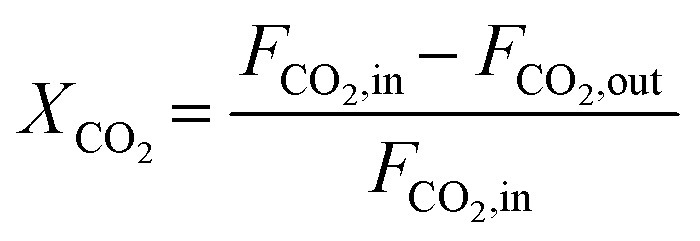
4
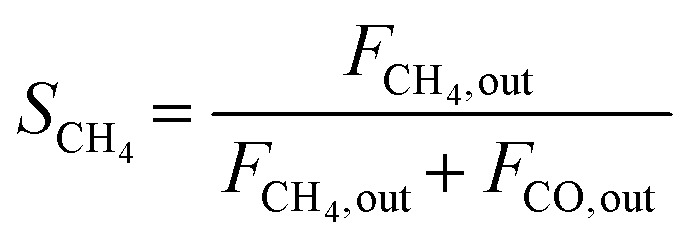
5
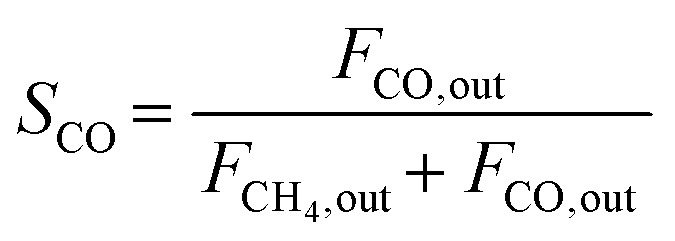

6 *Y*_CH_4__ = *S*_CH_4__·*X*_CO_2__

7
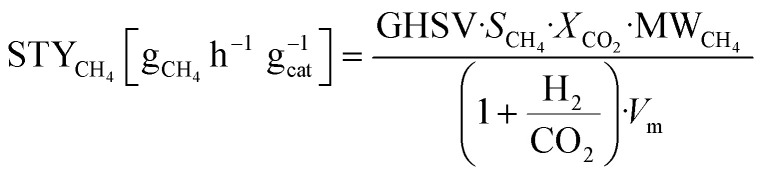
In eqn (7), the molar volume (*V*_m_) equals 22 400 cm^3^ mol^−1^ and the units of the gas-hourly space velocity (GHSV) and molar weight of methane (MW_CH_4__) are in cm^3^ h^−1^ g_cat_^−1^, and g_CH_4__ mol^−1^, respectively.

### 2.6. Active machine learning

Active learning is performed to explore and model a process with a minimal amount of experiments. The open-source algorithm Gaussian *n*-dimensional Active Learning Framework (GandALF) is used. This algorithm creates, when trained on labeled experimental data, a surrogate model for the objective using Gaussian processes. Then, the next experiment that meets informativeness, representativeness, and diversity criteria is identified using the expected model output change (EMOC) acquisition function. The working principle of the active learning framework is described in detail elsewhere.^38^

In the original implementation of the active learning framework, only one experiment is selected and performed at a time. This way of sequentially acquiring and performing experiments is not optimal for the objective of this study given the time-consuming aspect of synthesizing a selected catalyst. Therefore, we modified the code to overcome sequential single data selection so that it allows to select up to three different catalysts and three reaction conditions per catalyst, per iteration, in order to save up practical experimental time in the lab. The batch acquisition of next experiments allows up to nine activity tests to be performed per iteration. We refer to section S3 of the ESI† for the details about the active learning strategy.

### 2.7. Regression models

The dataset compiled using human-in-the-loop active learning was used to create a predictive model. Three kinds of supervised machine learning models for regression were investigated for this task: random forests (RF), extreme gradient boosting (XGB) regressors, and Gaussian processes (GP). All models were created in Python 3 using the open-source packages scikit-learn,^46^ xgboost,^47^ and GPy.^48^ Nested cross-validation was employed to tune the models and avoid overfitting. In the outer folds, the dataset is split into a training set and a test set (multiple outer folds). The training set is reduced in the inner folds into an inner training set and a validation set (multiple inner folds per outer fold). The best set of hyperparameters is chosen by the average performance over all inner folds. The model is evaluated for the outer fold by training the best model on the training set and testing it on the test set. This procedure is repeated for all outer folds. The prediction accuracy was calculated by the mean absolute error (MAE), the root mean squared error (RMSE), and the coefficient of determination (*R*^2^). Shapley additive explanation (SHAP) values^49^ were calculated for feature importance analysis and to provide local interpretability.

## 3. Results and discussion

### 3.1. Human-in-the-loop active learning CO_2_ hydrogenation study

Modeling a chemical process with satisfactory accuracy requires the development of a representative dataset from which information can be extracted. In active learning, similar to traditional design of experiments, the aim is to extract maximum information from minimum experiments. In particular, the objective is to learn trends arising from the intrinsic effect of variables within the design space and achieve high *X*_CO_2__ and *S*_CH_4__. To achieve fast information acquisition, an automated workflow is established that includes planning, executing, and analyzing catalytic experiments.

**Fig. 1:**
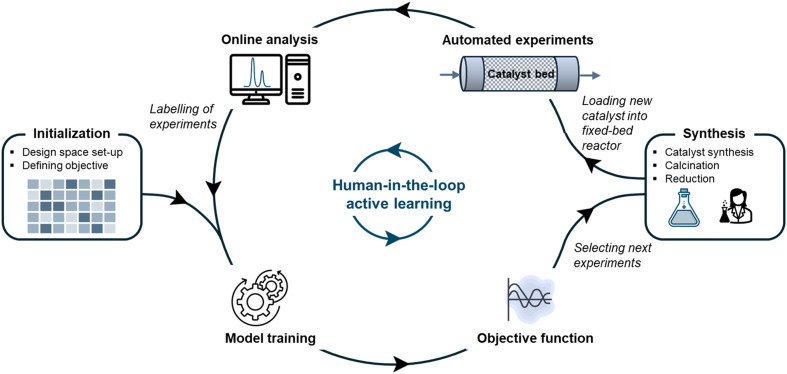
General workflow for human-in-the-loop active learning of a catalytic reaction.

The workflow of this study is illustrated in Fig. 1 and comprises six steps: (1) initializing the active learning algorithm, (2) training the algorithm, (3) selecting new experiments, (4) synthesizing catalysts, (5) performing catalytic activity tests, and (6) analyzing the activity tests. This workflow is a human-in-the-loop machine learning process in the sense that the researcher produces the catalysts and supervises the accuracy of experiments and analyses.

At first, the researcher sets up the experimental design space to be explored (see section 2.1) and defines the objective to be modeled. Since it is of interest to know the conversion, selectivity, and space–time yield, the methane yield (*Y*_CH_4__) was selected as the objective based on which experiments are suggested. *Y*_CH_4__, namely, contains information about *X*_CO_2__ and *S*_CH_4__ without the strong GHSV-dependence that defines STY_CH_4__. After the initialization, the researcher performed five experiments and the iterative process by training the Gaussian processes for the first time. From then on, new sets of experiments were iteratively suggested with the EMOC algorithm from the objective function. The manual steps are a sequence of synthesizing, drying, calcining, and reducing the catalyst, before reaction initialization in the fixed-bed reactor. The reaction conditions are controlled *via* LabVIEW and the raw analytical reaction output data that analyzes the product concentration at the reactor outlet is automatically processed into a format that allows retraining the machine learning model. The threshold was set at eight experimental rounds, resulting in a total of 26 synthesized catalysts and 48 performed catalytic tests.

**Fig. 2:**
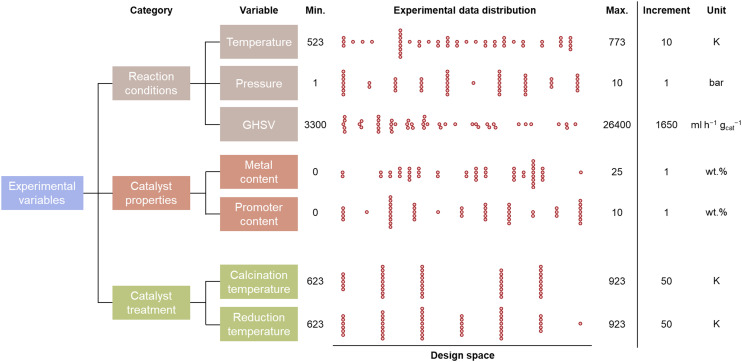
The distribution of the experimental variables in the acquisition of a catalytic activity training database, with the boundaries and step size of the experimental design space.

The distribution of the experimental variables over the training database that is created by human-in-the-loop active learning is shown in Fig. 2. It can be observed that data is spread evenly over the complete design space. However, the reaction conditions are more uniformly distributed than the catalyst properties and treatment. This is due to the fact that 48 distinctive reaction conditions were performed while only 26 catalysts were synthesized.

**Fig. 3:**
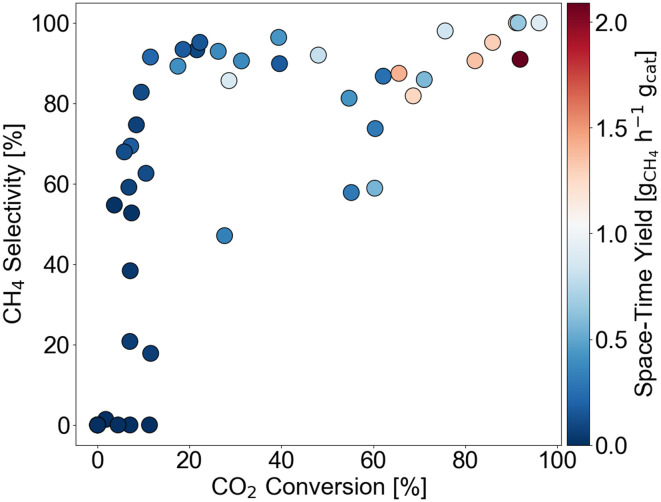
Output distribution of data points in the training set with respect to CO_2_ conversion, CH_4_ selectivity, and space–time yield.

The output distribution is given by Fig. 3 with respect to *X*_CO_2__, *S*_CH_4__, and STY_CH_4__. In terms of *X*_CO_2__ and *S*_CH_4__, the data is well-distributed despite a missing quadrant of high conversion-low selectivity catalysts. This missing quadrant is attributable to thermodynamic limitations. Namely, the CO_2_ methanation reaction is able to reach full conversion at these reaction conditions but the reverse water-gas-shift reaction is limited to lower conversions.^50^ When it comes to STY_CH_4__, bias towards STY_CH_4__ below 1 g_CH_4__ h^−1^ g_cat_^−1^ is observed.

### 3.2. Prediction of catalyst activity

#### 3.2.1. Model performance

The experimentally compiled training database with 48 samples is used to train supervised machine learning models in order to predict the catalytic activity of new samples. Additionally, an external test set of ten experimental conditions is randomly drawn from the experimental design space. These ten additional points are included to independently assess the predictive performance and generalizability of the trained model. Experiments were executed in the same manner as described above to annotate the ten external data points.

**Fig. 4:**
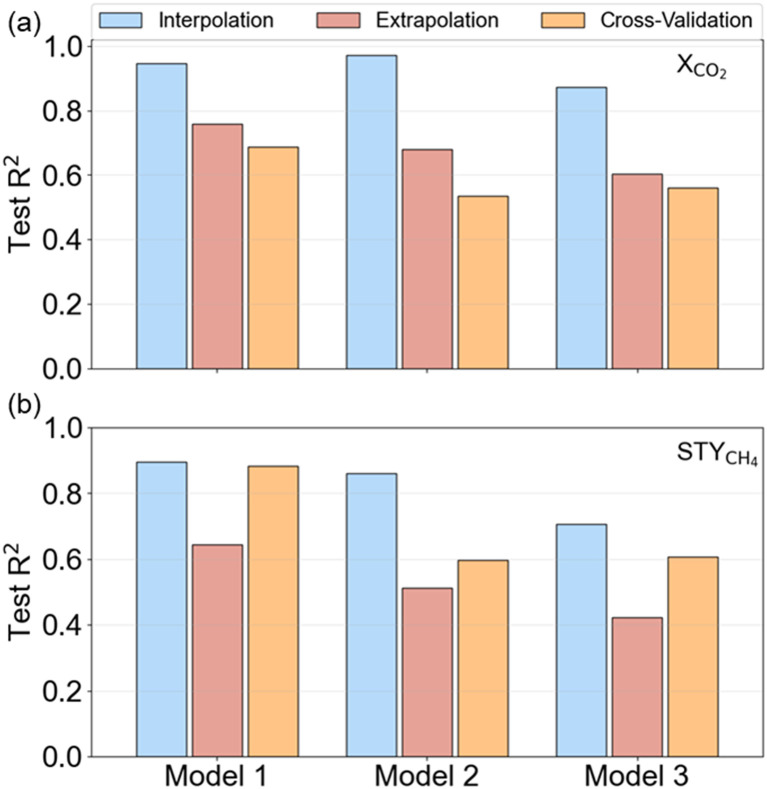
Machine learning model performance tested on different test sets. *R*^2^ of Gaussian processes (model 1), extreme gradient boosting (model 2), and random forests (model 3) on (a) *X*_CO_2__ and (b) STY_CH_4__ prediction.

Three supervised machine learning regression algorithms were employed to predict the *X*_CO_2__ and STY_CH_4__ for sets of experimental variables. Gaussian processes, which are the driving force behind the active learning workflow, are an obvious first choice. Next to Gaussian processes (GP, model 1), extreme gradient boosting (XGB, model 2), random forests (RF, model 3) and are evaluated. A stratified 8-fold cross-validation scheme with 4 repeats was used to cross-validate the model. Herein, we applied nested cross-validation in which the performance was evaluated on the outer folds. Hyperparameter optimization was performed solely on the training database and the best model was selected based on the average performance on the inner folds. Fig. 4a and b show the cross-validation accuracy of the three models on *X*_CO_2__ and STY_CH_4__ prediction, respectively, tested on an interpolation and extrapolation test set. The coefficient of determination (*R*^2^) was chosen as the performance metric. A higher *R*^2^ indicates a better predictive performance.

The model performance is measured on various test sets. An extrapolative test set was constructed out of ten samples, randomly drawn from the pool of potential experiments, with a low similarity to the training samples. The similarity is calculated using the Mahalanobis distance,^51^ following the same procedure (see ESI†) as in previous works.^52,53^ A test sample is in the interpolative range if its Mahalanobis distance to the center of the training set is smaller than the average distance over the whole training set. To construct an interpolation set, the 48 experiments from the training set and the external data points were combined to a large set of 58 data points. Ten samples that met the Mahalanobis distance criterion were then drawn from these 58 data points, whereby the 48 remaining samples were used as a new training set.

Fig. 4 illustrates the accuracies obtained with the different algorithms for *X*_CO_2__ and STY_CH_4__ prediction. It can be seen that GP and XGB achieve higher *R*^2^ values than the RF. Moreover, the scores for *X*_CO_2__ predictions are higher than for STY_CH_4__. This finding can be linked to the output data distribution, which is more imbalanced for STY_CH_4__ (see Fig. 3) and, hence, pose a more difficult prediction task. Overall, predictions are poorer on the extrapolation task, so that caution is required when relying upon model predictions. GPs achieve the highest *R*^2^ on the interpolative tests, respectively 0.95 and 0.90 for *X*_CO_2__ and STY_CH_4__. The repeated cross-validation results were found to be in the same order of magnitude as the interpolation and extrapolation sets, implying no overfitting. From these results, it is suggested that a decent initial guess of the catalytic activity can be made with a dataset as small as 48 samples. However, it is essential to use the model in the interpolative zone to obtain predictions with higher accuracy.

#### 3.2.2. Optimal catalyst design

We demonstrate that it is also possible to predict optimal sets of experimental variables with a machine learning model trained on a small catalytic dataset. For this task, we predicted the outcomes of a pool of 100 000 data points from the design space using the GP regression model in the active learning framework. Then, the data point is selected for which the posterior mean is maximized with *X*_CO_2__ and STY_CH_4__ as objectives. We did not consider *S*_CH_4__ as an objective as multiple points had already been found in the training set reaching a maximal *S*_CH_4__ of 100%. This approach reflects Bayesian optimization in which an explorative acquisition function is used for several rounds, followed by an extremum search with a fully exploitative acquisition function. Another strategy for optimal catalyst design would be to immediately make use of an optimization-oriented algorithm with an acquisition function that balances exploration and exploitation.

**Fig. 5:**
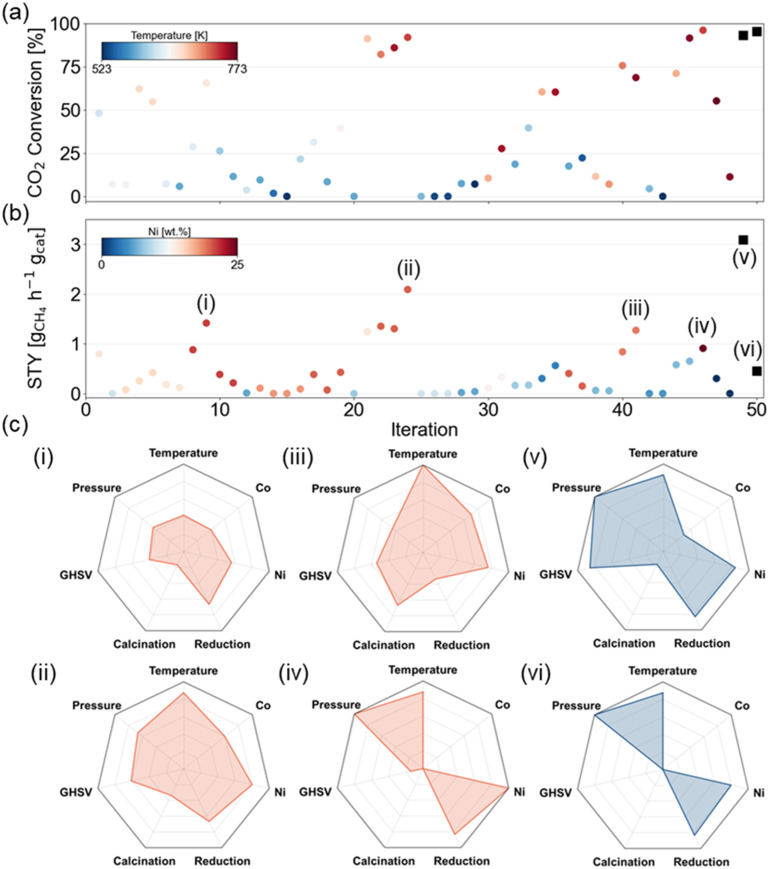
Overview of the (a) *X*_CO_2__ and (b) STY_CH_4__ for all experiments in the training set and the two selected optimal points (black squares). (c) Radar plots show the experimental details for 4 selected training samples (red, i–iv) and the two optimized experiments (blue, v–vi).

Fig. 5 shows the *X*_CO_2__ and STY_CH_4__ for each experiment that was picked by the active learning framework. Since this tool was not used as an optimization algorithm but rather as a screening tool in the training phase, an increasing trend in the objectives over the experiments is not expected. In terms of *X*_CO_2__, the currently measured maximum is 96.1% which is found in the 46th experiment. The room for improvement is limited to only a few percent, and this is reflected in the optimal experiment choice of the active learning framework. The set of experimental variables that should optimize *X*_CO_2__ is highly resembling to the earlier found maximum and with 95.6% the objective is not improved. The highest STY_CH_4__, on the other hand, amounts to 2.09 g_CH_4__ h^−1^ g_cat_^−1^ and was found in the 24th experiment in the training set. In this design space, the theoretically maximal STY_CH_4__ is 3.8 g_CH_4__ h^−1^ g_cat_^−1^ (at the highest GHSV, full conversion and selectivity) and the current optimum is only 55% of this aforementioned theoretically maximal STY_CH_4__. According to the model interpretations, the ideal set of experimental variables to maximize STY_CH_4__ consists of an elevated reaction temperature, pressure, and reduction temperature, a high GHSV and metal load, and a low to medium calcination temperature. The selected experiment from the active learning framework is a 21Ni–3Co/Al_2_O_3_ catalyst calcined at 673 K and reduced at 873 K, which is tested at 733 K and 10 bar with a GHSV of 23 100 ml h^−1^ g_cat_^−1^, which demonstrates that these trends are learned by the model itself. Indeed, in the experimental activity test, a STY_CH_4__ of 3.10 g_CH_4__ h^−1^ g_cat_^−1^ was observed. This observation indicates that the model-predicted set of experimental variables increased the STY_CH_4__ with nearly 50%.

### 3.3. Effect of experimental variables on reaction rate

#### 3.3.1. Feature importance analysis

The catalytic activity prediction models are black boxes that provide an output for a given set of experimental variables without explanations. In order to interpret how model predictions were obtained, we used the Shapley Additive Explanations (SHAP) method^54^ to investigate the influence of the individual experimental variables on the catalytic activity predictions with the XGB model. SHAP is an algorithm originating from game theory that adds interpretability to black-box model predictions. It does so by decomposing the predicted outcome into an additive function of individual feature contributions using Shapley values.^55^ The contribution of an individual feature can be calculated by normalizing the Shapley values. Higher normalized Shapley numbers indicate a larger contribution of an experimental variable to the model prediction.

**Fig. 6:**
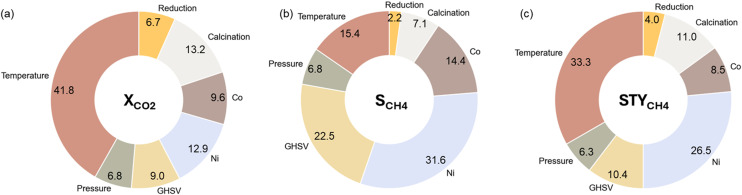
Relative importance based on Shapley Additive Explanations (SHAP) values of the considered experimental variables for (a) *X*_CO_2__, (b) *S*_CH_4__, and (c) STY_CH_4__.

Fig. 6 shows the relative impact of each experimental variable on the prediction of *X*_CO_2__, *S*_CH_4__, and STY_CH_4__. The repeated stratified 8-fold cross-validation approach from section 3.2.1 was utilized to make sure that the feature importance analysis holds over multiple train-test splits. The relative importance of a feature is thus the average importance over 32 trials. It is observed that temperature emerges as the most important experimental variable for *X*_CO_2__, where the reaction rate increases with temperature. However, thermodynamically, it is known that the lower temperatures are preferential for the methanation reaction. This is reflected in Fig. 6b, where the contribution of temperature is only 15.4% to the methane selectivity. Compared to thermal effects, the contribution of pressure is rather limited. The main metal load, in this case, Ni content, is another large driver of the catalytic activity. In *S*_CH_4__ prediction, Ni content has the largest contribution with a higher load having a positive effect on the selectivity.

The calcination temperature has a nonnegligible influence on the predicted *X*_CO_2__, *S*_CH_4__, and STY_CH_4__. In all three cases, increasing the calcination temperature within our range of 623 to 873 K has a negative effect on the output. This shows that a higher calcination temperature indicates a lower catalytic activity. However, it can be observed in the summary plots that an optimum temperature must be present for Ni–Co catalytic systems. The impact of the reduction temperature is limited, but higher reduction temperatures appear to have a beneficial impact on the catalytic activity, possibly due to the complete reduction of the active metallic species.

#### 3.3.2. Scientific interpretability of predicted trends

**Fig. 7:**
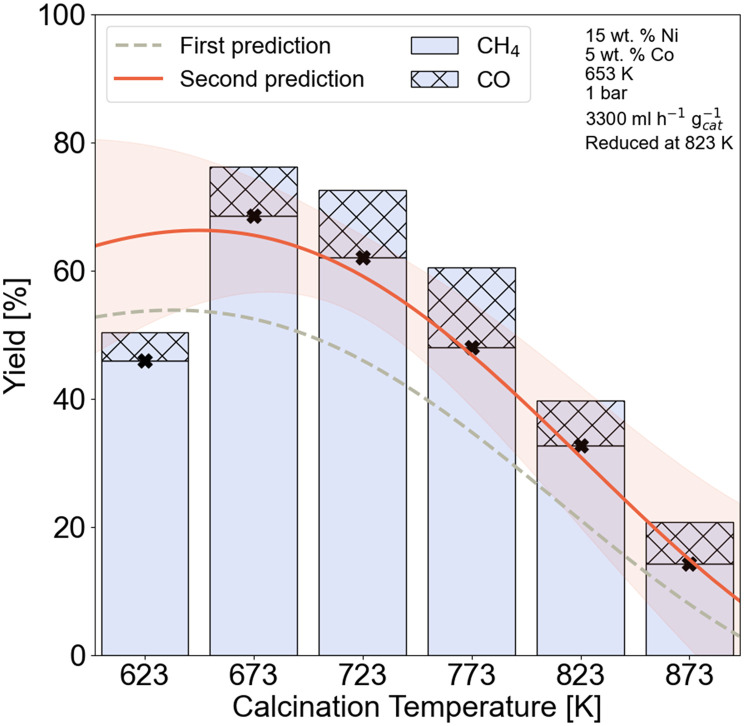
Effect of calcination temperature on the CH_4_ yield. Experimental conditions are given in the right inset. Dashed lines represent the first prediction of yield by the model, and red lines represent the second prediction. The orange band indicates the model uncertainty.

In the previous section, it was shown how much an experimental variable contributes to the catalytic activity. Apart from the importance of a feature, it is typically required to understand how and why a feature has an influence on the output. Since the effect of most experimental variables on the methanation reaction is well-studied,^56^ we demonstrate herein only how the calcination temperature affects the catalytic activity of Ni–Co/Al_2_O_3_ catalysts. To do so, we varied the calcination temperature for a representative catalytic system (15Ni–5Co/Al_2_O_3_), while keeping the remaining experimental variables constant. Based on the distance criterion introduced earlier, the experiments fall within the extrapolative range. In Fig. 7, the initially predicted trend with the GP surrogate model (model 1 in Fig. 4) is shown by the gray dashed line. Consequently, to bring the system into the interpolative range, one experiment was performed at the temperature with the highest uncertainty (723 K) and added to retrain the model. The newly predicted trend is shown in orange. It can be seen that the shape of the predicted trend does not change with additional experiments and corresponds to the trend witnessed in Fig. 6. However, the accuracy of the predicted methane yields improved significantly by only performing one additional experiment. A maximal methane yield and *X*_CO_2__ is witnessed around 673 K, while further increasing the calcination temperature has a detrimental effect on the catalytic activity of the 15Ni–5Co/Al_2_O_3_ sample.

We characterized the catalysts that were calcinated at various temperatures to investigate the effect of the calcination on the structure and the performance. The purpose of the calcination step in catalyst synthesis is to activate the catalyst so that the metal oxides are formed, which later can be followed by reduction to achieve the metal phase if needed. The method of the chosen calcination (calcination temperature, heating rate, gas flow, *etc.*) procedure often affects the final particle size.^57^

**Fig. 8:**
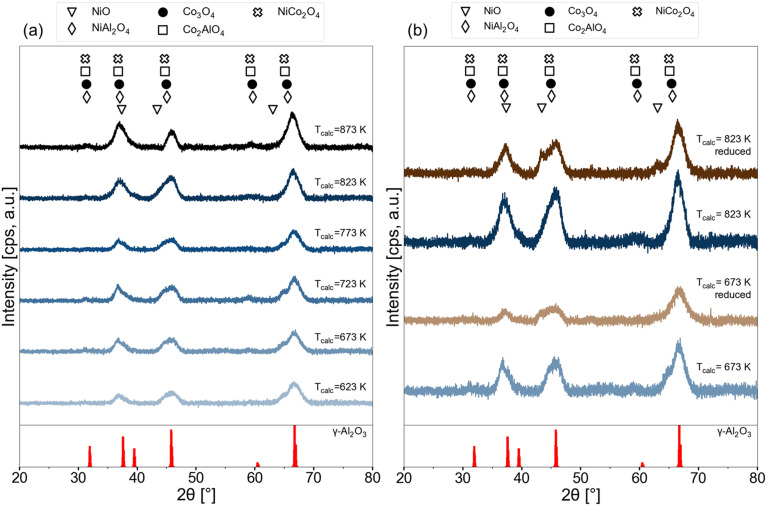
XRD patterns of 15Ni–5Co/Al_2_O_3_ samples (a) at various calcination temperatures (b) freshly calcined at 673 K and 823 K and reduced versions of these freshly calcined catalysts at 823 K.

XRD patterns of the 15Ni–5Co/Al_2_O_3_ samples calcined at different temperatures are given in Fig. 8a. Commonly, the diffractions of γ-Al_2_O_3_ (PDF 00-029-0063) are present in all of the samples. Some of the X-ray diffraction patterns of some oxides and aluminates of nickel and cobalt are difficult to distinguish from each other fully as they are very close to each other.^58,59^ The possible phases that were considered in this study were NiO (PDF 01-073-1519), NiAl_2_O_4_ (PDF 01-081-0715), Co_3_O_4_ (PDF 04-003-0984), CoAl_2_O_4_ (PDF 04-016-4504), NiCo_2_O_4_ (PDF 04-013-0797).

In the XRD patterns in Fig. 8a, broadening of the peak at around 36° is observed with increasing the calcination temperature, which indicates that the crystallite size is decreasing. With higher calcination temperatures, the intensity of the peaks that can be ascribed to the existence of the aluminates decreases. This weaker intensity can suggest that high calcination temperatures (above 823 K) treatments maintain lower crystallinity (higher degree of disorder, more of an amorphous structure) and/or higher dispersion of the spinel aluminate phases. After prolonged calcination at high temperatures (>500 K), the nickel species are reported to be stabilized against sintering by forming a nickel aluminate phase at the surface of the particles, as shown in eqn (8).^60,61^

8 NiO or CoO + Al_2_O_3_ → NiAl_2_O_4_ or CoAl_2_O_4_

Fig. 8b illustrates the XRD patterns of the 15Ni–5Co/Al_2_O_3_ samples after reduction at 823 K that were calcined at 673 and 823 K. The X-ray patterns of Ni, Co, and Ni–Co alloys are difficult to distinguish as they own very close diffraction peaks.^15^ However, the shift of the peak at about 46° towards a lower position upon reduction may indicate metal or alloy formation.^15^ The related intensities of NiO diffraction peaks become clearly visible after the reduction, possibly indicating NiO particles with bigger sizes. This shows that after reduction of 15Ni–5Co/Al_2_O_3_ samples, the relatively harder to reduce Ni^2+^ in NiAl_2_O_4_ spinel phase transforms to those in NiO and may also to metallic species.

Thermogravimetric analysis (TGA) and X-ray photoelectron spectroscopy (XPS) measurements were done to further investigate the effect of calcination temperature on the nickel species. The TGA results for the 15Ni–5Co/Al_2_O_3_ up to 973 K with a heating rate of 5 K min^−1^ are given in Fig. S3.2 and Table S3.2† illustrating no significant thermal events occurrence after 693 K. The XPS spectra for Ni 2p are given in Fig. S3.3† and the peak fitting ESCA parameters^62^ are given on Table S3.3.† It is seen from the Ni 2p spectra that the main surface Ni species belong to those of NiAl_2_O_4_ at calcination temperatures between 623 and 873 K. Nickel aluminates also seemed to be the main bulk phase as XRD confirmed. A full summary of the relative surface concentrations of NiAl_2_O_4_ and NiO, derived from the deconvoluted Ni 2p spectra is given in Table S3.4.† The relative surface concentration of NiAl_2_O_4_ increases from 89.48% at 623 K to 97.54% to 873 K, while the relative NiO surface concentration drops from 10.52% to 2.46%. However, the highest surface concentration of NiO belonged to the sample calcined at 673 K with 11.77%. This temperature-dependent variations in surface concentrations indicate that higher calcination temperatures strengthen the interaction between the γ-Al_2_O_3_ support and nickel species, hence lead to NiAl_2_O_4_ formation. On the other hand, lower calcination temperatures increase the relative NiO content. NiAl_2_O_4_ species are reported to be inactive and more difficult to be reduced in comparison with NiO.^63,64^ The relative increase in NiAl_2_O_4_ species can be correlated to the drop in CH_4_ yield with increasing calcination temperature of 15Ni–5Co/Al_2_O_3_ catalysts, as was observed in Fig. 7.

The highest BET surface area owning sample in our study was 15Ni–5Co/Al_2_O_3_, which was calcined at 673 K as given in Table S3.2.† As Table S3.5† shows, for the 15Ni–5Co/Al_2_O_3_ samples that were calcined higher than 673 K, we observed a slight decrease in the BET surface area with an increase in the calcination temperature.

**Fig. 9:**
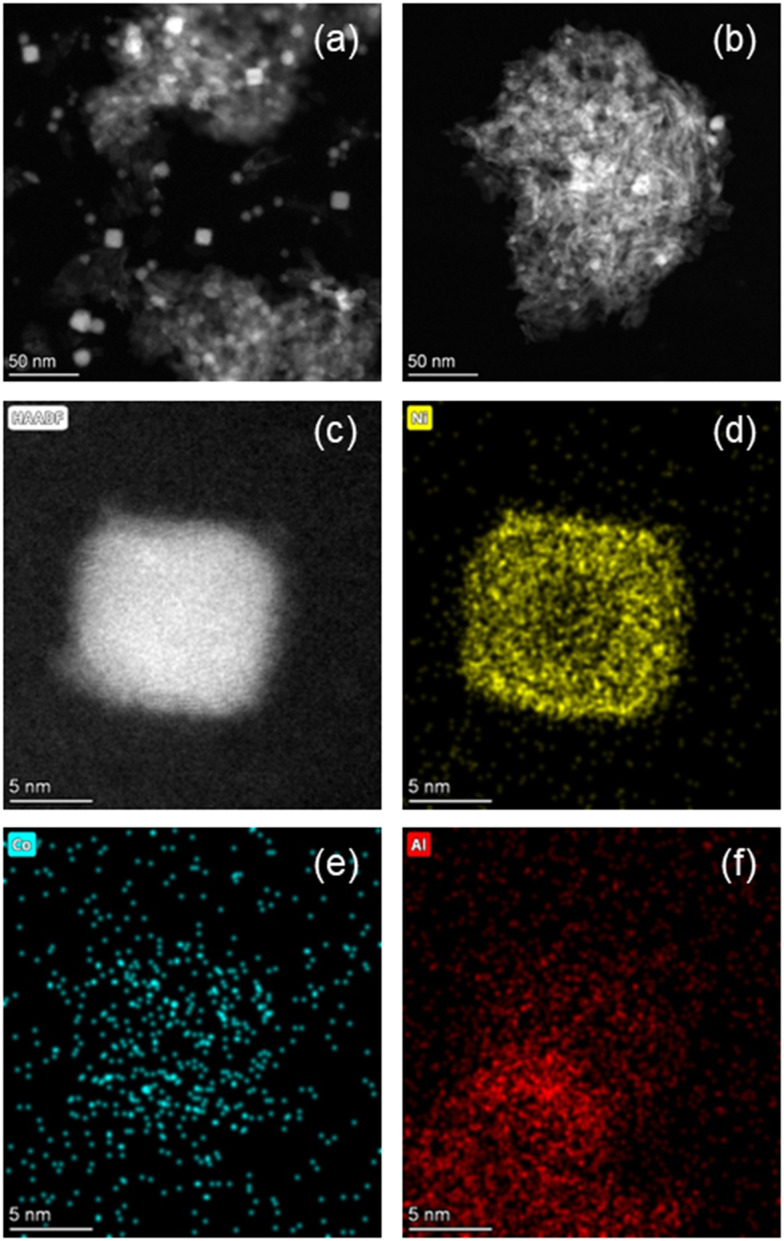
HAADF-STEM images of (a) reduced 15Ni–5Co/Al_2_O_3_-673 and (b) reduced 15Ni–5Co/Al_2_O_3_-823. (c) Magnified HAADF-STEM image of reduced 15Ni–5Co/Al_2_O_3_-673. Elemental mapping of reduced 15Ni–5Co/Al_2_O_3_-673 for (d) Ni, (e) Co, (f) Al.

The HAADF-STEM images of the 15Ni–5Co/Al_2_O_3_ catalysts that are calcined at 673 K and 823 K and both reduced at 823 K are shown in Fig. 9. The average nickel nanoparticle sizes of the reduced catalysts are 12.6 nm ± 1.2 nm for 15Ni–5Co/Al_2_O_3_-673 and 8.4 nm ± 1.8 nm for 15Ni–5Co/Al_2_O_3_-823. It was observed that the nickel particle size decreases with the increasing calcination temperature, which was in line with the XRD. Even though metal particle size decreases with calcination temperature increase, the metal nanoparticles seem rather to be located in clusters. This can be explained by the higher dispersion of the metal aluminate phases at higher calcination temperatures, as suggested by XRD. As Fig. 9 illustrates, the smaller metal nanoparticles are dispersed throughout bigger clusters which may influence the accessibility of the reactants to the active metal sites. Fig. 7 and section S5† already demonstrated the predicted detrimental effect of increasing calcination temperature on to *X*_CO_2__, *S*_CH_4__, and STY_CH_4__, which aligned with experimental catalytic activity tests. Evidently, lower to medium calcination temperatures presumably lead to higher BET surface areas. Furthermore, there are indications that these temperatures facilitate the reduction of metal species due to the weaker interaction with the support and that the metal species are better dispersed throughout sample but with bigger particle sizes.

In this section, we derived the understanding behind the combination of the reaction conditions and catalyst features that give superior yield that our active machine learning algorithm arrived to. The optimum point was able to be explained by surface and bulk characterizations. As simultaneous multi-dimensional changes are not easy-to-comprehend by humans, our methodology helps experimental researchers to speed up their work by providing a better start for optimizing their parameters with fewer experiments and characterizations amongst an immense chemical design space. Our strategy can save experimental time and cost which paves the way to discoveries in more complex reaction systems.

#### 3.3.3. Predictive kinetics with active learning

We evaluate that the utility of the active learning framework comprises both the investigation of synthesis and treatment parameters, as well as the screening of reaction conditions for individual catalysts. To this aim, we used the GP surrogate model to predict the light-off curve of a representative catalytic system. Such light-off curves show the conversion of a pollutant as a function of the reaction temperature for a specific catalyst at fixed operating conditions. In this case study, we investigated two catalytic systems. The first one is 19Ni–4Co/Al_2_O_3_, which was selected from the pool, calcined at 723 K, and reduced at 823 K. The second catalytic system is the 21Ni–3Co/Al_2_O_3_ that was selected in the previous section and resulted in the highest found STY_CH_4__. Both catalysts are tested at the default reactor conditions of 1 bar pressure and 3300 ml h^−1^ g_cat_^−1^ GHSV, and temperatures that vary between 523 K and 773 K.

**Fig. 10:**
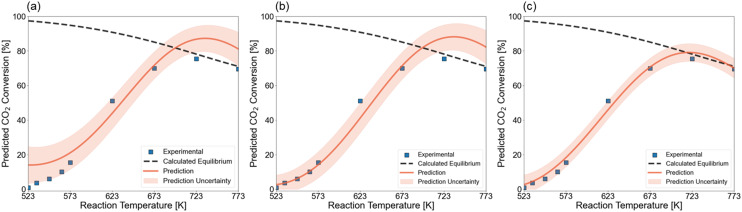
Light-off curve prediction for a 19Ni–4Co/Al_2_O_3_ catalyst, calcinated at 723 K, reduced at 873 K. The test is performed at 1 bar and 3300 ml h^−1^ g_cat_^−1^ GHSV. (a) Prior prediction without knowledge about the catalytic system. (b) Predicted curve after performing one experiment with the 19Ni–4Co/Al_2_O_3_ catalyst at the zone of highest uncertainty. (c) Posterior prediction in which the Gaussian processes model the performed activity tests.

In first instance, we predicted the light-off curve without any information about the tested catalytic systems. This prediction is shown in Fig. 10a and resulted in a light-off curve that follows the experimental data but lacks accuracy in the higher and lower temperature area. As such, an infeasible maximum *X*_CO_2__ is predicted that would be above the thermodynamic equilibrium. It has already been shown that accuracy is lower at the boundaries of the design space due to reduced data availability. To improve the prediction accuracy, one data point in the zone with the highest uncertainty was added to the training set. This point, which corresponds to a temperature of 523 K, adds confidence to the lower end of the temperature range. Fig. 10b depicts the light-off curve that is predicted with the GP after adding this one data point. It is observed that the trend in the low-conversion zone is now perfectly learned. Still, above 623 K the prediction does not change. In Fig. 10c, we illustrate the posterior prediction over the kinetic catalytic data. That is, the model is trained on the experimental data points and is able to model the trend without overfitting on the individual data points. Traditionally, researchers create power-law models or more detailed microkinetic models *via* numerous experiments to first calculate kinetic descriptors and then *a posteriori* simulate the effect of reaction conditions on the catalytic activity. Here, the model is capable of fitting experimental data accurately given a representative dataset and a minimal number of additional experiments.

## 4. Conclusion

In this study, we present a novel approach employing a human-in-the-loop workflow enhanced by active machine learning to evaluate the performance of Ni- and Co-based catalysts supported on Al_2_O_3_ for the thermo-catalytic conversion of CO_2_ into CH_4_. We assembled an experimental dataset comprising 48 catalytic activity tests conducted within a vast design space exceeding 50 million potential experiments. These tests were conducted utilizing an automated reactor system, ensuring systematic and controlled conditions throughout the experimentation process. The design space consisted out of variables that can be modified during the experimentation and/or during the catalyst synthesis, including the temperature, pressure, Ni and Co load, space velocity, calcination temperature, and reduction temperature. The compiled dataset was then used to train regression algorithms for predicting the CO_2_ conversion, methane selectivity, and methane space–time yield of new experiments. Three different regression algorithms were compared for the catalyst activity predictions: Gaussian processes, random forests, and extreme gradient boosting. It was found that Gaussian processes excelled in the interpolative range, while the extreme gradient boosting technique led to a more generalized model with the most accurate predictive performance for extrapolative tests. Feature importance analysis revealed that, next to temperature and Ni load, the calcination temperature also plays a crucial role for the activity of Ni–Co/Al_2_O_3_ catalysts in the methanation reaction. This finding was experimentally validated and it was seen that there is an optimal calcination temperature for Ni–Co/Al_2_O_3_ catalysts, lying between 673 and 723 K. Further increase of the calcination temperature is detrimental to the activity. Structurally, this was linked to the presence of oxide, aluminate and/or metallic species that were stable in the given calcination temperature. Our findings illustrate the efficacy of utilizing a modest database comprising only 48 catalytic tests to forecast a new set of experimental variables, resulting in a remarkable enhancement of methane space–time yield by nearly 50% compared to the highest value observed in the training set. Moreover, Gaussian processes exhibited exceptional accuracy in modeling the relationship between experimental variables and outcomes, enabling predictive kinetics provided the interpolation criterion is satisfied. Despite these advancements, interpreting black-box models remains a challenge. Nevertheless, the methodology outlined in this study, initially applied to the methanation reaction, can readily be adapted and transferred to address diverse chemical reactions with distinct design spaces.

## Data availability

The data supporting this article have been included as part of the ESI.† The entire source code is provided as open-source software under MIT license in the following repository: https://www.github.com/mrodobbe/gandalf-doe. All conclusions from the paper can be reproduced using the provided scripts. A demo notebook is available in the folder notebooks/demo.ipynb.

## Conflicts of interest

There are no conflicts of interest to declare.

## Supplementary Material

CY-014-D4CY00873A-s001
