# Supplementary material for: Accelerated design of nickel-cobalt based catalysts for CO2 hydrogenation with human-in-the-loop active machine learning
Source: Catal Sci Technol. 2024 Sep 10;14(21):6307–20. doi: 10.1039/d4cy00873a (PMC11391929; doi:10.1039/d4cy00873a)
Supplement: CY-014-D4CY00873A-s001 [file CY-014-D4CY00873A-s001.pdf]

Supplementary Information (SI) for Catalysis Science & Technology.
This journal is © The Royal Society of Chemistry 2024

Accelerated Design of Nickel-Cobalt Based Catalysts for CO_2 Hydrogenation with Human-in-the-Loop Active Machine Learning

Yasemen Kuddusi[1,2,*] , Maarten R. Dobbelaere[3], Kevin M. Van Geem[3], Andreas Züttel[1,2]

[1]Laboratory of Materials for Renewable Energy (LMER), Institute of Chemical Sciences and Engineering (ISIC), Basic Science Faculty (SB), École Polytechnique Fédérale de Lausanne (EPFL) Valais/Wallis, Energypolis, Rue de l'Industrie 17, 1951 Sion, Switzerland
[2]Empa Materials Science & Technology, 8600 Dübendorf, Switzerland
[3]Laboratory for Chemical Technology, Department of Materials, Textiles and Chemical Engineering, Ghent University, Technologiepark 125, 9052 Gent, Belgium

[*] Corresponding author: yasemen.kuddusi@epfl.ch

SUPPORTING INFORMATION

S1. Code Availability

The entire source code is provided as open-source software under MIT license in the following repository: https://www.github.com/mrodobbe/gandalf-doe. All conclusions from the paper can be reproduced using the provided scripts. A demo notebook is available in the folder notebooks/demo.ipynb.

S2. Hyperparameter Optimization

The key hyperparameters of each model were optimized via grid search. The optimized hyperparameters are marked in bold.

S2.1. Extreme Gradient Boosting

Extreme gradient boosting (XGB) models are created with the open-source software XGBoost (version 1.6.2) in python 3.7. The following XGB hyperparameters are optimized via grid search:

- Number of estimators: [500, 1000, 2500, **5000**, 10000]
- Maximum depth: [**2**, 3, 4]
- Learning rate: [0.001, 0.005, 0.01, 0.025, 0.05, 0.1, 0.25, 0.5, **0.75**]
- Subsample: [0.05, 0.1, 0.25, 0.5, 0.6, 0.7, 0.75, **1**]
- Tree method: [**histograms**, exact]

S2.2. Random Forests

Random forests (RF) regressors are implemented via scikit-learn (version 1.3.2 in python 3.11). The following RF hyperparameters are optimized via grid search:

- Number of estimators: [**100**, 500, 1000]
- Maximum depth: [None, 2, **5**]

- Maximum features: [**sqrt**, log2]
- Minimal samples per split: [**2**, 5, 10]

S2.3. Gaussian Processes

Gaussian process regression (GP) is implemented via GPy (version 1.12 in python 3.7). The following GP hyperparameters are optimized via grid search:

- Kernel: [Rational quadratic, exponential, linear, Matérn 32, Matérn 52, **radial basis function**]

S3. Gaussian n-dimensional Active Learning Framework (GandALF)

The Gaussian n-dimensional Active Learning Framework (GandALF) was applied in this study and in this section, we repeat the working principle of this algorithm [1].

GandALF is a versatile design-of-experiments tool that can handle continuous, discrete, and categorical variables. It selects the next experiment or set of experiments based on informativeness, representativeness, and diversity. The informativeness criterion imposes that an experiment should be chosen so that it adds information to the model while minimizing the information redundancy. An experiment is representative when it follows the distribution of experiments and is not an outlier. The diversity is guaranteed when experiments are selected that are sufficiently different from each other. Before initiating the experiments, a pool of P unlabeled data points is drawn from the design space. Here, the pool size P is kept at the default size of 100,000. The initial experiments are selected by clustering the pool of potential experiments into n_{init} (in this work: n_{init} = 5) initial experiments using the k-means algorithm [2]. The centroid of every cluster is picked as an initial experiment. The selection of the centroid ensures that the most

representative experiment is chosen, hence ensuring the representativeness of the initial experiments.

A Gaussian process with a radial basis function (RBF) kernel is used as surrogate model. The Gaussian process surrogate model is created a first time using the initial experiments, after which GandALF chooses new experiments. From the second iteration on, a new pool is drawn from the design space with P equal to $20{,}000 \times n$, with n the number of previously performed experiments. Then, this pool is divided in $n + 1$ clusters. The selection process solely considers experiments from the largest $n_{cluster}$ empty clusters. The original GandALF version was fully sequential and, therefore, $n_{cluster}$ was equal to 1. In this work, $n_{cluster}$ is set to 3. We define an empty cluster as a cluster that only contains unlabeled, potential experiments. The choice of the largest clusters ascertains that the representativeness criterion is met. Since these clusters are empty, the diversity criterion is satisfied. Finally, the next $n_{experiments}$ experiments are drawn from each picked cluster. Again, as GandALF was originally fully sequential, $n_{experiments}$ was equal to 1, meaning that 1 experiment was drawn from the single largest empty cluster. In this work, $n_{experiments}$ is set to 3, whereby the constraint is added that the catalyst properties and catalyst treatment are fixed, which restricts the diversity criterion. Thus, a maximum of 9 experiments (i.e., $n_{cluster} \times n_{experiments}$) can be selected per iteration. The next experiments are selected from the empty clusters using the expected model outcome change (EMOC) acquisition function [3]. The use of the EMOC acquisition function is to ensure that the experiment is informative, as the acquisition function requires an accurate determination of the uncertainty of the outcome for a specific experiment.

S4. Experimental Details

S4.1. Experimental Set-up

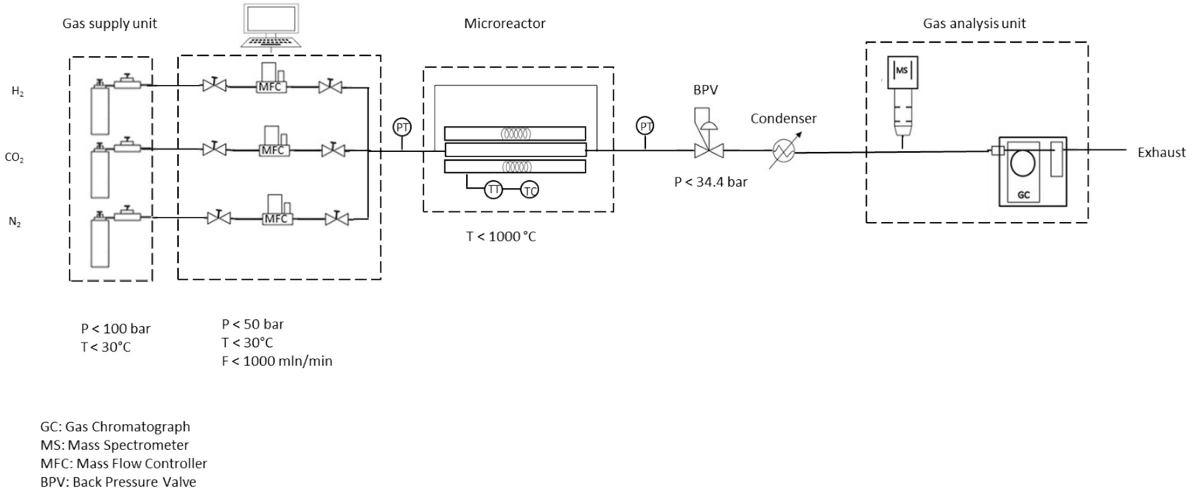

Figure S3.1. Plan of the experimental setup with gas supply and controlling unit, the microreactor and the gas analysis unit.

S4.2. Catalytic Activity Tests

Table S3.1. Overview of all experiments performed in this work with the experimental variables and output metrics.

T [K]	P [bar]	GHSV [ml/h/g]	Ni [%]	Co [%]	T calc [K]	T red [K]	Y CH_4 [%]	X CO_2 [%]	S CH_4 [%]	S CO [%]	STY CH_4 [g/h/g_{cat}]
623	5	12552	14	4	673	823	44.19	48.05	91.97	8.03	0.79
643	5	16375	10	5	873	823	0.00	7.05	0.00	100.00	0.00
643	5	13141	15	5	823	673	4.03	6.81	59.11	40.89	0.08
673	1	3300	15	0	723	773	53.92	62.17	86.73	13.27	0.25
673	1	6598	15	5	723	773	44.47	54.73	81.25	18.75	0.42
633	9	25024	14	10	873	673	5.00	7.21	69.35	30.65	0.18
583	7	20332	14	10	873	673	3.95	5.82	67.90	32.10	0.12
633	7	25070	21	10	673	623	24.49	28.60	85.63	14.37	0.88

663	9	17236	21	10	673	623	57.34	65.58	87.44	12.56	1.42
603	10	10968	21	10	673	623	24.40	26.26	92.91	7.09	0.38
583	10	14102	21	10	673	623	10.53	11.51	91.49	8.51	0.21
623	1	3271	6	1	623	673	2.00	3.65	54.67	45.33	0.01
583	8	9474	18	2	823	723	7.87	9.51	82.75	17.25	0.11
553	4	11053	18	2	823	723	0.02	1.78	1.38	98.62	0.00
523	4	7895	18	2	823	723	0.00	0.00	0.00	0.00	0.00
613	7	3158	18	2	823	723	20.15	21.60	93.28	6.72	0.09
633	3	9410	20	9	873	873	28.35	31.31	90.54	9.46	0.38
583	1	7841	20	9	873	873	6.31	8.45	74.64	25.36	0.07
653	5	7841	20	9	873	873	37.98	39.41	96.37	3.63	0.43
583	8	16154	8	2	673	673	0.00	0.00	0.00	0.00	0.00
683	4	9532	14	0	623	673	91.15	91.15	100.00	0.00	1.24
713	2	12692	20	6	723	823	74.39	82.13	90.58	9.42	1.35
753	3	11106	20	6	723	823	81.78	85.96	95.13	4.87	1.30
733	7	17452	20	6	723	823	83.61	91.95	90.92	9.08	2.09
593	4	19412	10	3	823	723	0.00	0.00	0.00	0.00	0.00
533	8	24265	10	3	823	723	0.00	0.00	0.00	0.00	0.00
523	1	16176	10	3	823	723	0.00	0.00	0.00	0.00	0.00
583	3	3283	6	7	873	723	3.90	7.40	52.72	47.28	0.02
523	6	9850	6	7	873	723	2.73	7.11	38.35	61.65	0.04
693	2	11379	13	2	873	823	6.60	10.54	62.58	37.42	0.11
753	1	17882	13	2	873	823	13.01	27.64	47.07	52.93	0.33
583	8	6567	8	3	673	773	17.37	18.60	93.38	6.62	0.16
603	5	3284	8	3	673	773	35.57	39.59	89.85	10.15	0.17
693	7	4737	4	7	873	823	44.53	60.42	73.70	26.30	0.30
753	8	11052	4	7	873	823	35.51	60.31	58.88	41.12	0.56
583	3	18060	20	6	673	723	15.60	17.48	89.22	10.78	0.40
543	10	4925	20	6	673	723	21.17	22.25	95.14	4.86	0.15
683	7	21029	7	2	873	673	2.06	11.56	17.80	82.20	0.06
703	5	25882	7	2	873	673	1.46	7.03	20.76	79.24	0.05
713	7	7895	19	7	823	723	74.12	75.65	97.98	2.02	0.84
763	5	15789	19	7	823	723	56.21	68.69	81.84	18.16	1.27
593	4	21557	5	3	723	623	0.00	4.45	0.00	100.00	0.00
523	1	9950	5	3	723	623	0.00	0.00	0.00	0.00	0.00
693	8	6600	7	8	723	773	61.06	71.10	85.89	14.11	0.58
763	10	4950	7	8	723	773	91.53	91.53	100.00	0.00	0.65

733	10	6600	25	0	623	873	96.08	96.08	100.00	0.00	0.91
763	1	6600	0	10	623	923	31.91	55.21	57.79	42.21	0.30
763	9	8250	0	0	623	873	0.00	11.30	0.00	100.00	0.00
753	4	3300	25	1	623	823	91.10	91.10	100.00	0.00	0.43
643	4	23100	11	3	723	673	4.56	7.98	57.08	42.92	0.15
693	7	16500	19	6	873	823	71.08	71.08	100.00	0.00	1.68
743	5	14850	8	6	773	923	68.48	78.71	87.00	13.00	1.46
563	10	8250	22	7	673	723	14.07	14.07	100.00	0.00	0.17
753	1	16500	16	2	823	823	36.06	58.40	61.75	38.25	0.85
643	7	18150	17	0	723	723	68.31	68.31	100.00	0.00	1.78
563	3	8250	0	9	873	823	2.20	3.71	59.27	40.73	0.03
683	5	16500	23	4	873	923	84.73	84.73	100.00	0.00	2.00
613	6	11550	2	7	673	723	1.28	7.60	16.79	83.21	0.02
733	10	23100	21	3	673	873	93.28	93.28	100.00	0.00	3.09
733	10	3300	20	0	623	873	95.36	95.36	100.00	0.00	0.45

S4.3. TGA Curves

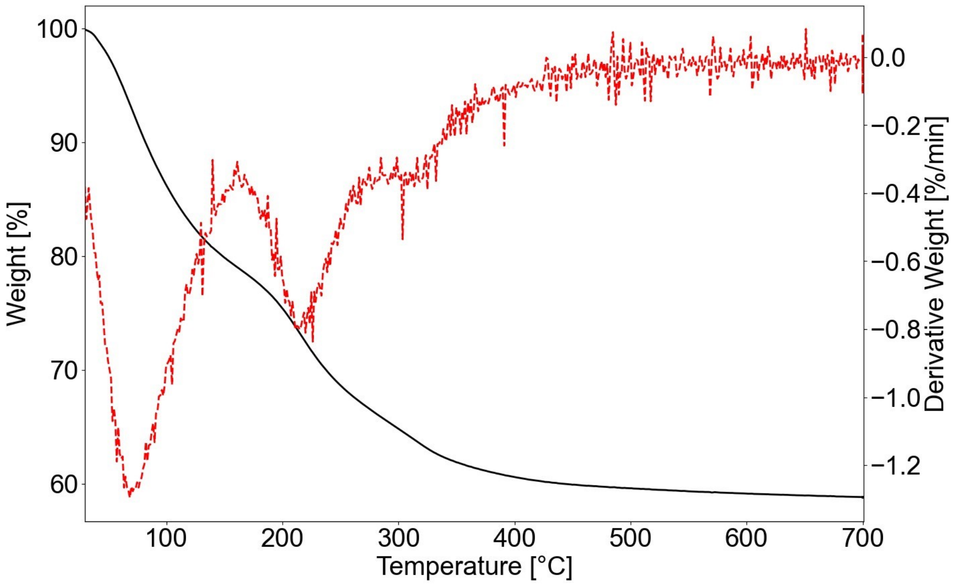

Figure S3.2. Thermogravimetric analysis (TGA) of 15Ni-5Co/Al_2O_3 under air flow heating up to 700°C with a heating rate of 5°C/min. Residual mass curve is represented by the black line and

differential residual mass curve is represented by the red line.

Table S3.2. Mass residuals and changes retrieved from TGA of 15Ni-5Co/Al_2O_3 under air flow heating up to 700°C with a heating rate of 5°C/min.

Temperature (°C)	Mass change from 30 °C (%)	Residual mass from 30 °C (%)
350	38.70	61.90
400	39.37	60.61
450	40.05	59.93
500	40.36	59.62
550	40.63	59.35
600	40.83	59.16
650	40.99	58.98
700	41.13	58.85

S4.4. XPS Spectra

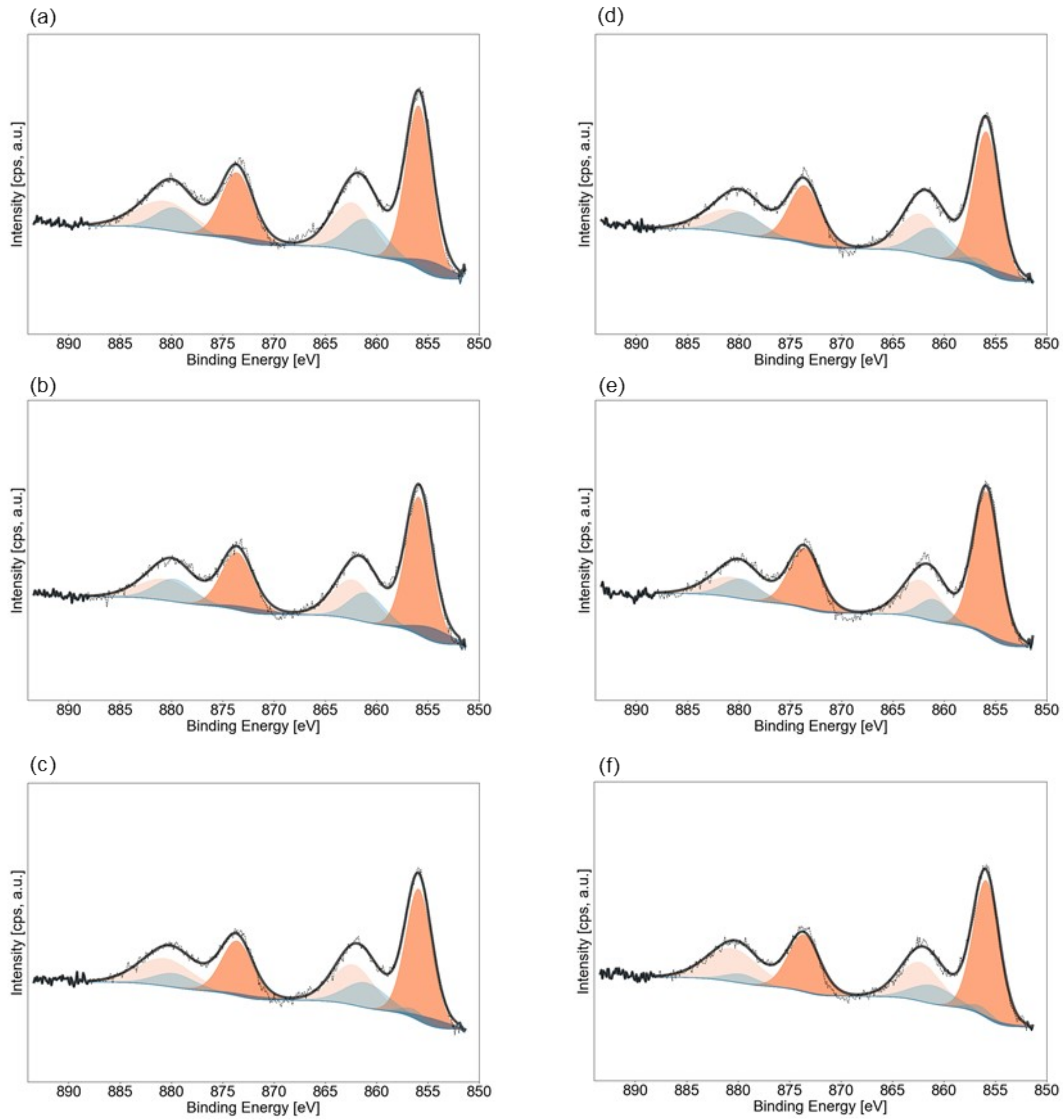

Figure S3.3. Ni 2p XPS spectra of (a) c623 (b) c673 (c) c723 (d) c773 (e) c823 (f) c873. Orange species represent $NiAl_2O_4$ and blue species represent NiO.

Table S3.3. ESCA parameters of Ni 2p lines. XPS fitting details for NiO and $NiAl_2O_4$ for main and satellite peaks. These parameters were retrieved from [4].

	**Ni $2p_{3/2}$**				**Ni $2p_{1/2}$**			
	Main peak		Satellite 1	Satellite 2	Main peak		Satellite 1	Satellite 2
	E_b	FWHM	E_b	E_b	E_b	FWHM	E_b	E_b
NiO	854.5	4.1	856.4	861.0	872.3	5.4	874.2	879.7
$NiAl_2O_4$	856.0	2.8	858.1	862.3	873.6	3.6	875.7	880.6

Table S3.4. NiO percentages throughout the samples that are calcined at different temperatures.

**Sample Coding**	**NiO / (NiO+$NiAl_2O_4$) at Ni $2p_{3/2}$ (%)**	**NiO / (NiO+$NiAl_2O_4$) at Ni $2p_{1/2}$ (%)**
c623	10.52	10.51
c673	11.77	11.78
c723	7.715	7.715
c773	6.590	6.590
c823	3.857	3.857
c873	2.458	2.458

S4.5. Textural Properties

Table S3.5. BET/BJH analysis results. Surface area, average pore volume and average pore size for $15Ni\text{-}5Co/Al_2O_3$ samples that are calcined at various temperatures.

Sample Coding	BET surface area (m^2g^{-1})	BJH pore volume (cm^3g^{-1})	BJH pore diameter (nm)
c623	193.69	0.4950	9.7454
c673	199.08	0.5347	10.263
c723	197.89	0.5546	10.649
c773	192.35	0.5570	11.102
c823	193.50	0.5434	10.625
c873	192.79	0.5570	10.915

S5. Mahalanobis Distance Criterion

The Mahalanobis distance (d_M) measures the distance between a distribution of data points and a single data point. In this case, the distance is measured between a set of experimental variables and the training set. It is calculated via eq (S1).

$$d_M^2 = z^T \Lambda'^{-1} z \quad \text{(S1)}$$

In eq (S1), z is a column vector containing the seven experimental variables and Λ' is the 7×7 covariance matrix of the experimental variables in the dataset.

S6. SHAP Values

The importance of a feature in a model can be visualized using summary plots. These plots sort the features vertically by importance. In each row, the SHAP value per data point is given. The color of this dot indicates the value of the feature. For example, a red dot in the temperature row indicates a set of experimental variables that includes a high temperature value. Figures S5.1 to S5.3 show the summary plots that are created for XGB models trained on CO_2 conversion, CH_4 selectivity, and CH_4 space-time yield. These summary plots were created using a single model of which the hyperparameters were optimized with stratified k-fold cross-validation. The training sets were the same of the interpolative model (48 data points collected via active learning).

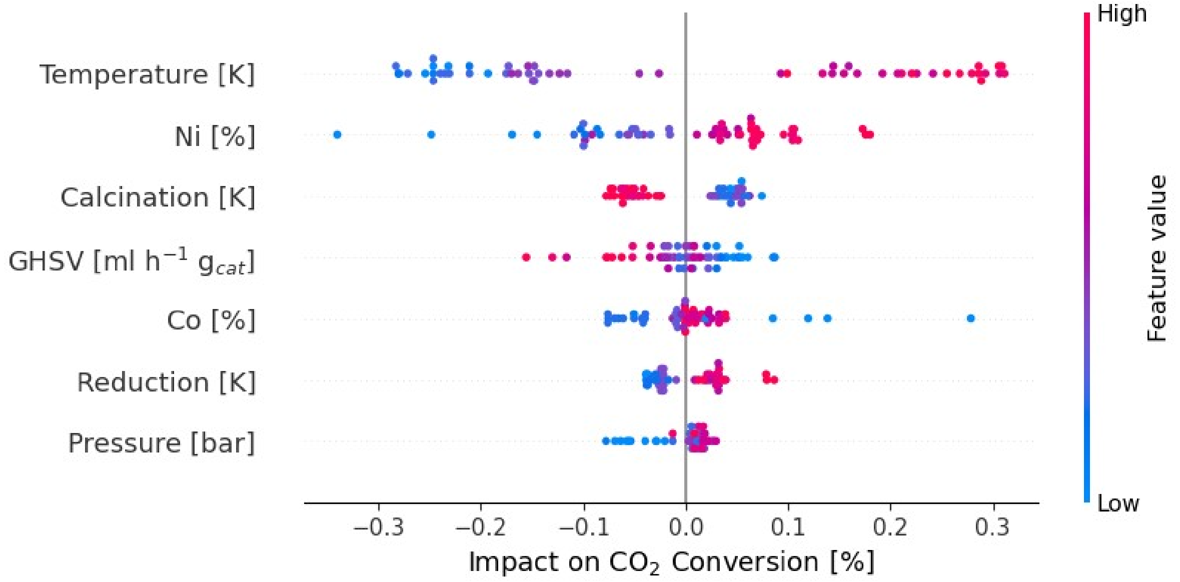

Figure S5.1. Summary plot between experimental variable and the impact on the CO_2 conversion.

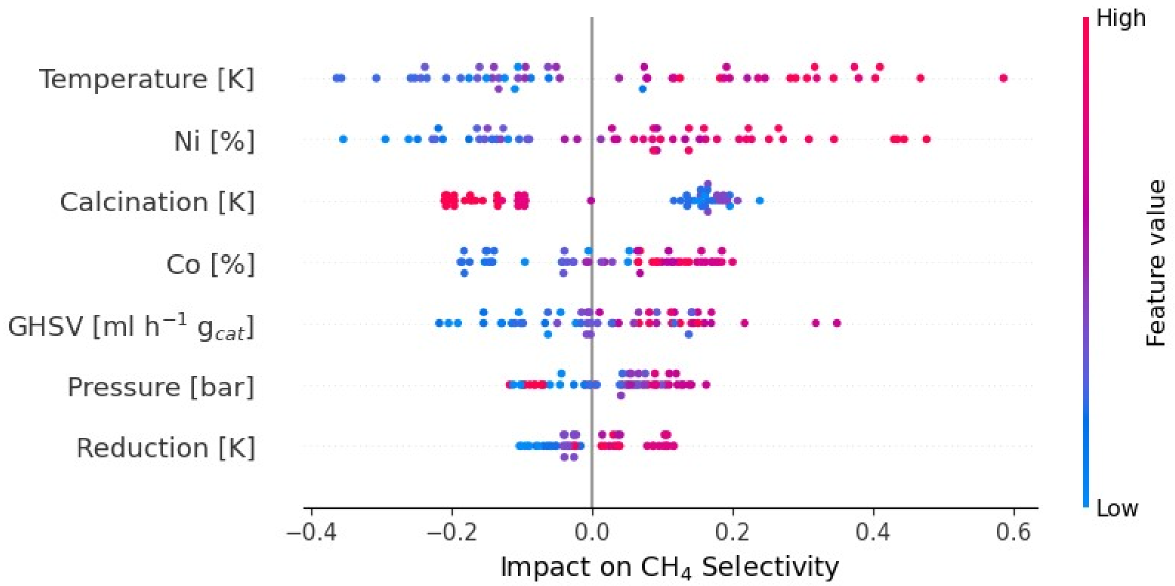

Figure S5.2. Summary plot between experimental variable and the impact on the CH_4 selectivity.

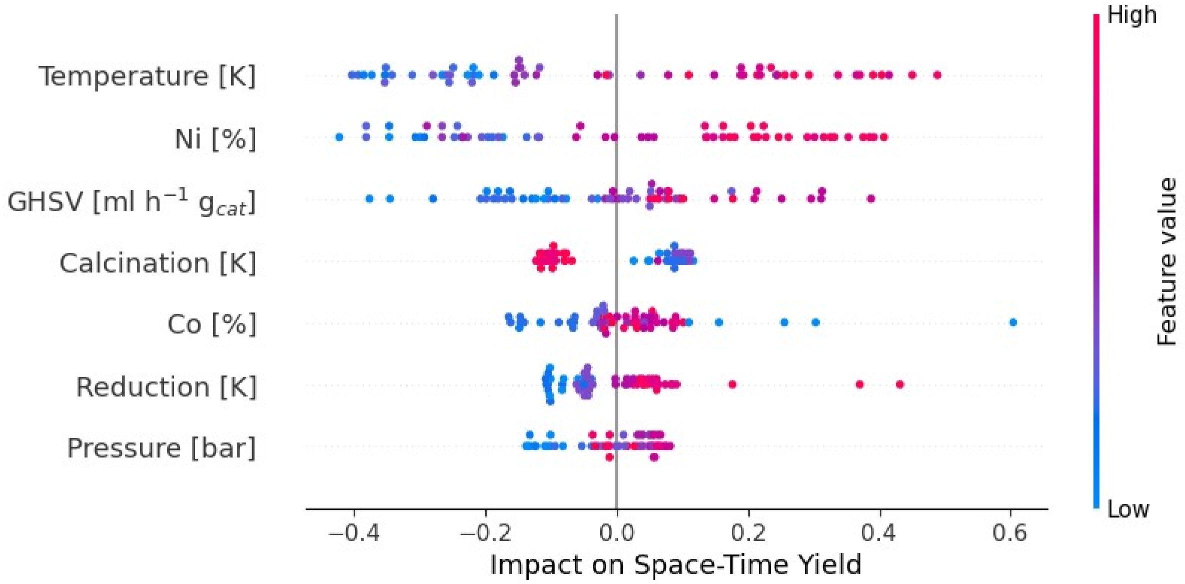

Figure S5.3. Summary plot between experimental variable and the impact on the CH_4 space-time yield.

S7. Validation by Kinetic Modeling

A microkinetic model is directly interpretable and it provides a relationship between the catalyst structure and its performance. A trade-off lies in the prediction of kinetic parameters. To this extent, we make use of the CO_2 methanation modeling assumptions set by Mutschler *et al.* [5]. Here, we create a model to validate the obtained results.

The rate law that was derived in the work of Mutschler *et al.* [5] was used to calculate the activation energy and Arrhenius parameters. Below is a short overview of the equations required to derive the activation energy.

The rate is defined as the change in partial pressure of CO_2, as shown in equation (S2).

$$-\frac{dp_{CO_2}}{dt} = r(t) \tag{S2}$$

We derive the rate law for the kinetically limited zone, in which we assume that the backward reaction is close to zero. We define the rate law in equation (S3), in which k_+ is the forward rate coefficient, p_{CO_2} is the partial pressure of CO_2, p_{H_2} is the partial pressure of H_2, and α and β are the reaction order with respect to CO_2 and H_2.

$$r(t) = k_+ p_{CO_2}^{\alpha} p_{H_2}^{\beta} \tag{S3}$$

The Arrhenius equation (equation (S4)) relates k_+ with the pre-exponential factor A, the activation energy E_A, the ideal gas constant R, the temperature T.

$$k_+ = A\exp\left(-\frac{E_A}{RT}\right) \tag{S4}$$

Mutschler *et al.* [5] further assume that because of the stoichiometry of the reaction and the corresponding change in partial pressure (equation (S5)), the reaction orders of CO_2 and H_2 are linked.

$$\Delta P_{CO_2} = \frac{\Delta P_{H_2}}{4} \tag{S5}$$

With the relationship between the partial pressures, the reaction rate can be rewritten as given in equation (S6).

$$r(t) = k_+ p_{CO_2}^{\alpha} \left(4p_{CO_2}\right)^{4\alpha} = k'_+ p_{CO_2}^{5\alpha} \tag{S6}$$

To determine the reaction order, the reaction rate is rewritten as a function of the space velocity (SV) and the conversion of CO_2 molecules (X_{CO2}) (equation (S7)).

$$r(t) = SV \cdot \left(1 - X_{CO_2}\right) = k"_+ p_{CO_2}^{\alpha} \tag{S7}$$

The natural logarithm of equation (S7) allows to derive the reaction order α, as shown in equation (S8).

$$\ln\left(1 - X_{CO_2}\right) + \ln SV = \alpha \cdot \ln p_{CO_2} + \ln k"_+ \tag{S8}$$

To obtain the Arrhenius parameters, equation (S6) is rearranged to equation (S9) and integrated from the inlet partial pressure $p_{CO_2,i}$ to the outlet partial pressure $p_{CO_2,o}$ and from 0 to the residence time t_0.

$$\int_{p_{CO_2,i}}^{p_{CO_2,o}} \frac{dp_{CO_2}}{p_{CO_2}^{5\alpha}} = \int_0^{t_0} k'_+ \, dt \tag{S9}$$

The expression for the modified forward rate constant k'_+ is then obtained in equation (S10).

$$\frac{\left(1 - X_{CO_2}\right)^{1 - 5\alpha}}{1 - 5\alpha} = - k'_+ t_0 + C \tag{S10}$$

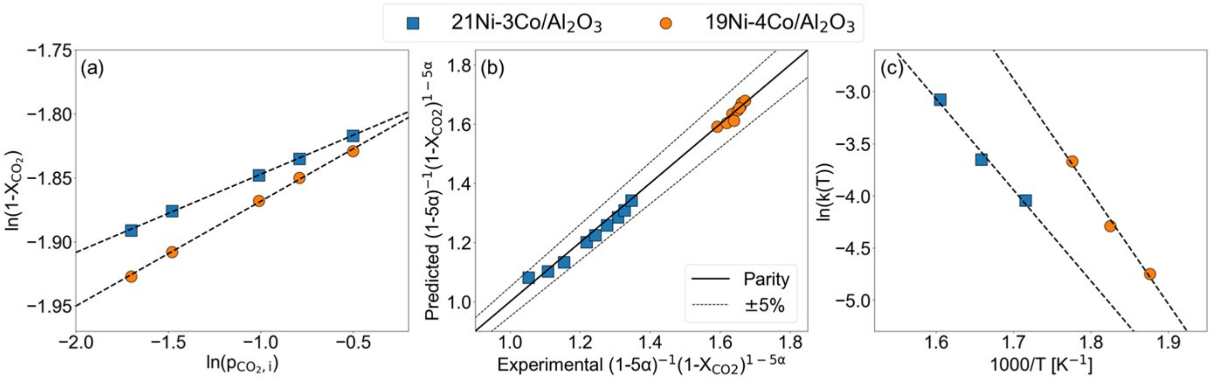

Figure S6.4. Experiments to derive the kinetic parameters of 19Ni-4Co/Al2O3 catalyst, calcinated at 723 K, reduced at 873 K (orange) and 21Ni-3Co/Al2O3 catalyst, calcinated at 673 K, reduced at 823 K (blue). (a) Linear regression to determine the reaction order. (b) Parity plot for the prediction of the integrated rate law values with Gaussian processes. (c) Linear regression of experimental rate constants with respect to temperature to determine the activation energy.

A first requirement is the experimental determination of the reaction order α which was given in equation (S8). This parameter cannot be estimated since the H_2/CO_2 ratio is kept constant in the design space. The reaction order for CO_2 is determined from the slope of the linear fits in **Figure S6.4.** a and found to be 0.08 for the 19Ni-4Co/Al_2O_3 and 0.06 for the 21Ni-3Co/Al_2O_3. In the modeling approach used here, which assumes a fractional rate law, the next step consists of deriving the Arrhenius parameters. This is done by varying the residence time at different temperatures. The rate coefficient is in this rate-law model determined by plotting the integrated rate law versus the residence time. The y-axis values are predicted using the model trained on the CO_2 conversion, X_{CO_2}. To ensure the interpolative regime and ascertain accuracy, one measurement is added to the training set at each different GHSV. It is seen in Figure S6.4. b that high accuracy (R^2>0.98) is achieved and the agreement between model and experimental data is in all cases within the 5% error bars. Nevertheless, to determine the Arrhenius parameters, a linear trend has to be obtained and despite the low absolute error, the model is too noisy extract these Arrhenius parameters. Experimentally, the activation energies are obtained with a highly linear trend, as shown in Figure S6.4. c. It was found that the 19Ni-4Co/Al_2O_3 sample has an activation energy of approximately 89 kJ/mol, while the 21Ni-3Co/Al_2O_3 sample has a lower activation energy of 73

kJ/mol. These values are in the order of magnitude of the activation energies that are found for similar catalytic systems, ranging between 70 and 100 kJ/mol [6], [7], [8], [9].

S8. Light-Off Curve

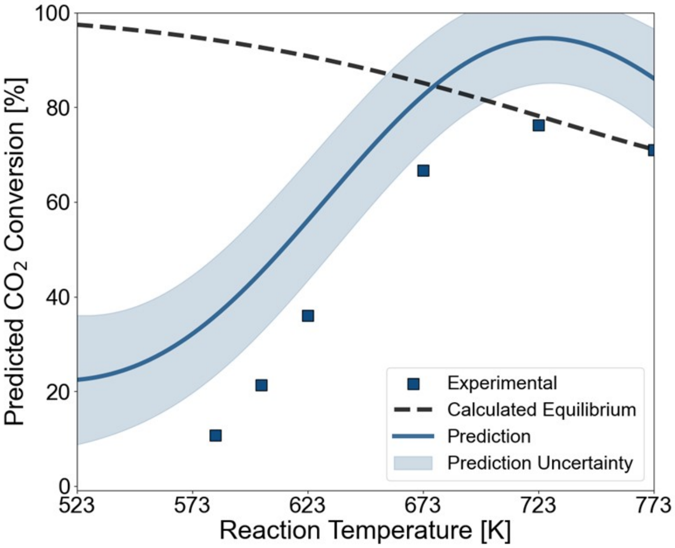

Figure S7.1. Prior light-off curve prediction without knowledge about the catalytic system for a 21Ni-3Co/Al_2O_3 catalyst, calcinated at 673 K, reduced at 873 K.

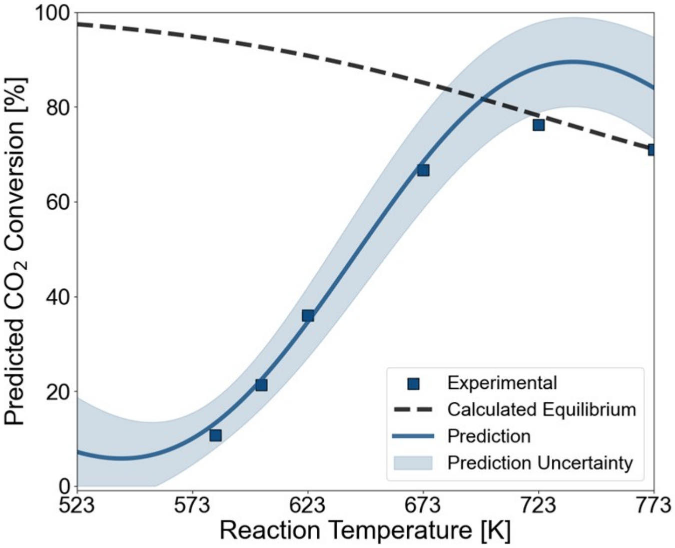

Figure S7.2. Predicted light-off curve after performing one experiment with the 21Ni-3Co/Al_2O_3 catalyst, calcinated at 673 K, reduced at 873 K.

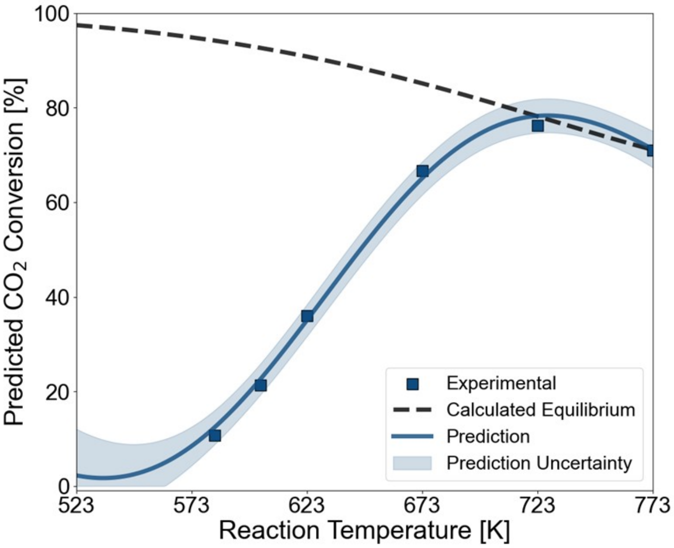

Figure S7.3. Posterior light-off curve prediction for the 21Ni-3Co/Al_2O_3 catalyst, calcinated at 673 K, reduced at 873 K after performing activity tests.

S9. References

[1] Y. Ureel *et al.*, "Active learning-based exploration of the catalytic pyrolysis of plastic waste," *Fuel*, vol. 328, p. 125340, Nov. 2022, doi: 10.1016/j.fuel.2022.125340.

[2] J. A. Hartigan and M. A. Wong, "Algorithm AS 136: A K-Means Clustering Algorithm," *Journal of the Royal Statistical Society. Series C (Applied Statistics)*, vol. 28, no. 1, pp. 100–108, 1979, doi: 10.2307/2346830.

[3] A. Freytag, E. Rodner, and J. Denzler, "Selecting Influential Examples: Active Learning with Expected Model Output Changes," in *Computer Vision – ECCV 2014*, D. Fleet, T. Pajdla, B. Schiele, and T. Tuytelaars, Eds., Cham: Springer International Publishing, 2014, pp. 562–577.

[4] C. P. Li, A. Proctor, and D. M. Hercules, "Curve Fitting Analysis of ESCA Ni 2p Spectra of Nickel-Oxygen Compounds and Ni/Al2O3 Catalysts," *Appl. Spectrosc.*, vol. 38, no. 6, pp. 880–886, Nov. 1984.

[5] R. Mutschler, E. Moioli, and A. Züttel, "Modelling the CO2 hydrogenation reaction over Co, Ni and Ru/Al2O3," *Journal of Catalysis*, vol. 375, pp. 193–201, Jul. 2019, doi: 10.1016/j.jcat.2019.05.023.

[6] A. Quindimil *et al.*, "Intrinsic kinetics of CO2 methanation on low-loaded Ni/Al2O3 catalyst: Mechanism, model discrimination and parameter estimation," *Journal of CO2 Utilization*, vol. 57, p. 101888, Mar. 2022, doi: 10.1016/j.jcou.2022.101888.

[7] T. A. Le, J. Kim, J. K. Kang, and E. D. Park, "CO and CO2 methanation over Ni/Al@Al2O3 core–shell catalyst," *Catalysis Today*, vol. 356, pp. 622–630, Oct. 2020, doi: 10.1016/j.cattod.2019.09.028.

[8] G. Garbarino *et al.*, "A study of Ni/La-Al2O3 catalysts: A competitive system for CO2 methanation," *Applied Catalysis B: Environmental*, vol. 248, pp. 286–297, Jul. 2019, doi: 10.1016/j.apcatb.2018.12.063.

[9] G. Garbarino, D. Bellotti, P. Riani, L. Magistri, and G. Busca, "Methanation of carbon dioxide on Ru/Al2O3 and Ni/Al2O3 catalysts at atmospheric pressure: Catalysts activation, behaviour

and stability," *International Journal of Hydrogen Energy*, vol. 40, no. 30, pp. 9171–9182, Aug. 2015, doi: 10.1016/j.ijhydene.2015.05.059.
